# Diversity of Metal—Fullerene Framework Structures Regulated by Metal Salts

**DOI:** 10.3390/nano12081314

**Published:** 2022-04-12

**Authors:** Jingjing Wang, Yang-Rong Yao, Shaoting Yang, Xinyi Zhou, Ao Yu, Ping Peng, Fang-Fang Li

**Affiliations:** 1 State Key Laboratory of Materials Processing and Die & Mould Technology, School of Materials Science and Engineering, Huazhong University of Science and Technology, Wuhan 430074, China; Jjwangup@163.com (J.W.); hust_yst@163.com (S.Y.); zhou99xy@163.com (X.Z.); aoyuhust@hust.edu.cn (A.Y.); 2 Department of Chemistry, University of Texas at El Paso, El Paso, TX 79968, USA; yyr880821@163.com

**Keywords:** fullerene ligand, coordination, metal salts, metal–fullerene framework, crystal structures

## Abstract

Taking into account the diversity of fullerene ligands and metal salts, metal–fullerene frameworks (MFFs) present a variety of structures. Currently, the structural control of MFFs mainly relies on the design and synthesis of fullerene ligands, while the influence of metal building units on the structures has been rarely studied. The present work represents a systematical investigation of fullerene-linked supramolecular architectures incorporating different metal salts. Treatment of a bidentate N,N-donors fullerene ligand (**L1**) with six metal salts ([Zn(NO_3_)_2_·6H_2_O, Cd(NO_3_)_2_·4H_2_O, Cu(NO_3_)_2_·3H_2_O, Cu(OAc)_2_·H_2_O, FeCl_2_·4H_2_O and FeCl_3_·6H_2_O]) produced six one-dimensional MFFs, i.e., Zn**L1**(NO_3_)_2_(H_2_O)_2_ (**1**), Cd**L1**(NO_3_)_2_ (**2**), Cu(**L1**)(H_2_O)_2_(NO_3_)_2_ (**3**), Cu**L1**(OAc)(CH_3_O) (**4**), Fe**L1**Cl_2_ (**5**) and Fe**L1**Cl_2_(FeCl_4_) (**6**). Compounds **1–3**, built with nitrates with different metal centers (M(NO_3_)_2_, M = Zn, Cd, Cu), present a 1D stair-like, 1D zigzag, and 1D linear chain structure, respectively. Compound **4**, synthesized with another Cu(II) salt, Cu(OAc)_2_, displays a dinuclear Cu-Cu connected 1D stair-like chain structure, rather than the single Cu linked 1D linear chain obtained from Cu(NO_3_)_2_. Compounds **5** and **6**, assembled from iron chloride of different oxidation states (Fe(II)Cl_2_ and Fe(III)Cl_3_) reveal a 1D zigzag and a 1D stair-like chain structure, respectively. The results demonstrate the significant influences of metal salts on the structures of metal–fullerene frameworks.

## 1. Introduction

Fullerene–metal compounds, represented by endohedral metallofullerenes [1,2], exohedral metallofullerenes [3,4,5,6], and metal–fullerene frameworks [7,8,9,10,11], are attractive due the synergistic and complementary characteristics of each moiety in terms of structure, properties, and potential applications [12,13,14,15]. The metal–fullerene framework (MFF) is similar to the metal–organic framework (MOF), except that fullerene molecules are used as building blocks in MFF, instead of the conventional organic ligands in MOF. Moreover, fullerenes with nanoscale size have the potential to form porous structures with large void spaces accessible for guest molecules.

The first fullerene–metal coordination compound was reported in 1998 by Diederich and co-workers, in which the reaction of a dipyridylmethanofullerene with Pt(Et_3_)_2_(OTf)_2_ formed a dimeric fullerene–Pt complex [16]. Although fullerenes modified with terpyridine, bipyridine, dipyrrolidine and pyrazine have been designed and synthesized, the coordination of these fullerene ligands with metal centers only produces discrete coordination complexes [17,18,19,20,21]. The first one-dimensional (1D) MFF was successfully prepared in 2007, wherein a fullerene ligand with two flexible pyridyl donors was synthesized and coordinated with metal ions [7]. Through the precise design of the molecular structures of fullerene ligands, a variety of 1D metal–fullerene frameworks have been realized [22,23,24]. MFF took a step forward in 2014 by achieving two-dimensional (2D) MFFs, in which a *trans*-1 fullerene ligand with two pairs of biphenylpyridine groups was designed as a building unit. This framework displays two types of large pores due to the large-sized fullerene ligand [8]. 2D MFF was also realized through the reaction of a bis-piperazine fullerene ligand with Rh_2_(O_2_CCH_3_)_4_ [25], which also exhibited large cavities. Three-dimensional (3D) supramolecular structures based on fullerene ligands are interesting organic frameworks and are expected to have potential applications. Beuerle and co-workers designed and synthesized a series of fullerene dodecaacid adducts, which served as building units for assembly with Zn, Cu, Cd and Ca metal centers to form 3D metal–fullerene frameworks [9,10].

Based on the above studies, great effort has been devoted to the design and synthesis of various fullerene ligands in order to tune the framework structures. However, the regioselective synthesis of symmetric fullerene ligands with high yields remains a great challenge due to the multiplicity of reactive bonds of fullerene C_60_ and the presence of multiple isomers. Metal salt, although receiving less attention, is also an integral part of metal–fullerene framework structures. The diversity of metal salts should bring about great opportunities to build various infinite structures via assembly with multidentate fullerene ligands. Nevertheless, the tuning capability of metal salts to the coordination mode of fullerene linkers and the metal–fullerene framework structures has rarely been studied. Considering the abundance of metal salts with different cations, anions and oxidation states, the regulation of metal salts on MFF structures deserves further study, which will lead to a variety of possibilities in building metal–fullerene framework structures.

Herein, six metal salts are selected for assembly with two fullerene ligands, one bidentate N,N-donors ligand (**L1**) and one bidentate N,O-donors ligand (**L2**). Unexpectedly, **L2** shows low coordination ability to the metal centers, and no supramolecular structures are obtained from **L2**. In contrast, six new MFF structures are successfully synthesized from **L1**, namely Zn**L1**(NO_3_)_2_(H_2_O)_2_ (**1**), Cd**L1**(NO_3_)_2_ (**2**), Cu(**L1**)(H_2_O)_2_(NO_3_)_2_ (**3**), Cu**L1**(OAc)(CH_3_O) (**4**), Fe**L1**Cl_2_ (**5**) and Fe**L1**Cl_2_(FeCl_4_) (**6**). **L1** and all the obtained MFFs are characterized by single-crystal X-ray diffraction. The influences of metal cations (*Zn*(NO_3_)_2_, *Cd*(NO_3_)_2_, *Cu*(NO_3_)_2_), counter anions (Cu(*NO_3_*)_2_ and Cu(*OAc*)_2_), and metal valence states (*Fe(II)*Cl_2_ and *Fe(III)*Cl_3_) on the structures of MFFs are demonstrated. This systematic study illustrates that in addition to the fullerene ligands, the structures of MFFs can be tuned by metal salts, providing valuable information for future elaboration of metal–fullerene frameworks. Moreover, it enriches the reservoir of the crystalline metal–fullerene frameworks.

## 2. Materials and Methods

Solvents and reagents were obtained from commercial sources and used without further purification. All synthesized crystals were characterized by single-crystal X-ray diffraction at 100K. The NMR spectra were recorded using a JEOL 600 NMR spectrometer. The mass spectrum was conducted on a Bruker autoflex speed mass spectrometer.

**Synthesis of fullerene ligands L1 and L2**: **L1** was synthesized according to our previous report [23]. The yellow crystals of **L1** were prepared by slow diffusion of methanol into a solution of **L1** in dichloromethane after a period of one month.

**Scheme 1:**
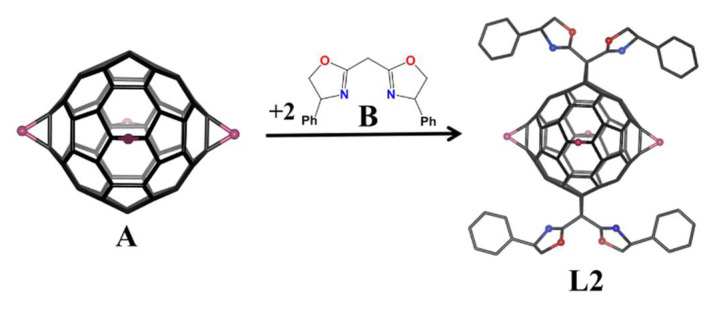
Synthesis of the *trans*-1 bis[(4S)-4-phenyl-2-oxazoline] adduct of C_60_-tetramalonate, **L2**, where R (purple balls) are the malonate groups, C(OOC_2_H_5_)_2_, blue balls are nitrogen atoms and red balls are oxygen atoms.

The synthesis of **L2** is shown in Scheme 1. Tetrakis-[di(ethoxycarbonyl)methao]-C_60_ (**A**) [23] and 2,2′-Methylenebis[(4S)-4-phenyl-2-oxazoline] (**B**) were synthesized according to the previous reports [26]. First, 67 mg (0.444 mmol) 1,8 diazabicyclo[5.4.0]undec-7-ene (DBU) was added to a CH_2_Cl_2_ solution of 100.0 mg (0.073 mmol) **A**, 73.5 mg (0.222 mmol) CBr_4_ and 67.9 mg (0.222 mmol) **B**. The reaction mixture was stirred at room temperature for 12 h. Then, the solvent was removed under reduced pressure and the product was purified by Prep-TLC to get light yellow solid **L2** (35 mg, 28% yield). Data for **L2**: ^1^H NMR (600 MHz, CDCl_3_, TMS): *δ* = 7.34 (m, 20H), 5.44 (t, 4H, *J* = 9 Hz), 4.80 (t, 4H, *J* = 9.6 Hz), 4.37 (dd, 8H, *J*_12_ = 7.2 Hz, *J*_13_ = 14.4 Hz), 4.31 (m, 12H), 1.34 (t, 12H, *J* = 7.2 Hz), 1.31 ppm (t, 12H, *J* = 7.2 Hz). ^13^C NMR (150 MHz, CDCl3): δ = 164.0, 163.6, 161.1, 146.2, 145.9, 145.8, 145.7, 142.2, 141.8, 141.6, 141.2, 141.1, 141.0, 128.7, 127.6, 127.1, 75.6, 70.3, 69.6, 69.2, 69.1, 62.9, 62.8, 45.6, 45.4, 34.7, 14.2, 14.1 ppm. NMR spectra of **L2** can be found in the Supplementary Information (Figures S1 and S2). MS calcd. C_126_H_72_O_20_N_4_, [M+H]^+^ = 1961.47; found 1961.76.

**Synthesis of compounds 1**–**6:** First, 0.5 mg **L1** was dissolved in 0.5 mL dichloromethane (CH_2_Cl_2_) in a glass tube, then 0.1 mL dichloromethane/methanol (1:1 *v*/*v*) mixed solution was carefully added into the glass tube as a buffer layer. After that, 1 mL methanol (CH_3_OH) solution of metal salts (20 equiv.) was discreetly added. The glass tube was sealed and kept undisturbed at room temperature. Crystals suitable for single-crystal X-ray diffraction were obtained after a month. The photographs of crystals can be found in Figure S3 in the supporting information.

**Synthesis of ZnL1(NO_3_)_2_(H_2_O)_2_ (1): L1** (0.5 mg, 1 equiv.) was dissolved in 0.5 mL dichloromethane in a glass tube, then 0.1ml dichloromethane/methanol (1:1 *v*/*v*) mixed solution was carefully added as a buffer layer. After that, 1 mL methanol solution of Zn(NO_3_)_2_·6H_2_O (1.76 mg, 20 equiv.) was added on the buffer layer. The glass tube was sealed and left undisturbed. Yellow crystals of **1** suitable for single-crystal X-ray diffraction were obtained after a period of a month.

**Synthesis ofCdL1(NO_3_)_2_ (2):** Yellow crystals of **2** suitable for single-crystal X-ray diffraction were obtained by following the same procedures as described for **1**, except for using Cd(NO_3_)_2_·4H_2_O (1.83 mg, 20 equiv.) as the metal source.

**Synthesis ofCuL1(H_2_O)_2_(NO_3_)_2_ (3):** Green crystals of **3** suitable for single-crystal X-ray diffraction were attained by following the same procedures as described for **1**, except for using Cu(NO_3_)_2_·3H_2_O (1.43 mg, 20 equiv.) as the metal building unit.

**Synthesis ofCuL1(OAc)(CH_3_O) (4):** Green crystals of **4** were obtained by following the same procedures as described for **1** except for using Cu(OAc)_2_·H_2_O (1.19 mg, 20 equiv.) as the metal salt.

**Synthesis ofFeL1Cl_2_ (5):** Green crystals of **5** were obtained by following the same procedures as described for **1** except for using FeCl_2_·4H_2_O (0.75 mg, 20 equiv.) as the building unit.

**Synthesis ofFeL1Cl_2_(FeCl_4_) (6):** Red crystals of **6** were prepared by following the same procedures as described for **1** except for using FeCl_3_ (0.96 mg, 20 equiv.) as the metal source.

**Single-Crystal XRD Measurements of L1 and Compounds 1**–**6.** Experimental data for the X-ray analyses are described in the Supplementary Materials. Single-crystal X-ray diffraction data of **1** were collected at 100 K in beamline station BL17B at Shanghai Synchrotron Radiation Facility. Single-crystal X-ray diffraction data of **L1** and **2**–**6** were collected on an XtaLAB PRO MM007HF diffractometer with Cu-Kα radiation (λ = 1.5406 Å). The CrystalClear software package (Rigaku) was used for data collection, cell refinement, and data reduction. Crystal structures were solved by the intrinsic phasing method and refined using full-matrix least-squares based on F^2^ with the programs SHELXT-2014 and SHELXL-2018 within OLEX2, respectively [27]. All of the non-hydrogen atoms were refined anisotropically, and the positions of the hydrogen atoms were generated geometrically. The SQUEEZE program [28] was used to remove the contributions of disordered solvents. CCDC 1878371, 1878374, 1878376–1878379 and 1881730 contain the supplementary crystallographic data for this paper. These data can be obtained free of charge via www.ccdc.cam.ac.uk/data_request/cif (accessed on 30 December 2021), or by emailing data_request@ccdc.cam.ac.uk, or by contacting The Cambridge Crystallographic Data Centre, 12 Union Road, Cambridge CB2 1EZ, UK; Fax: +44 1223 336033.

## 3. Results

**Figure 1:**
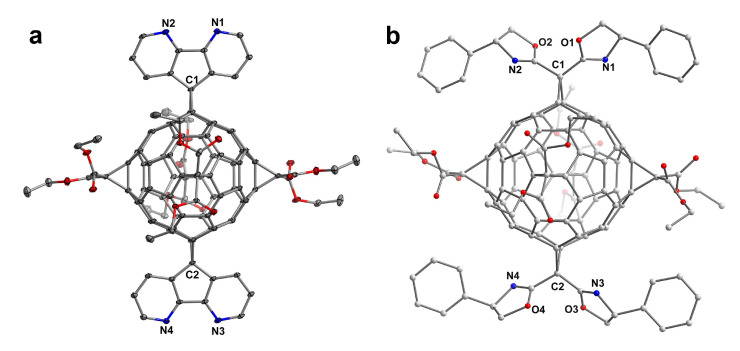
(**a**) Single-crystal structure of fullerene linker **L1** with 15% thermal ellipsoids, and (**b**) the molecular structure of **L2**. Hydrogen atoms were omitted for clarity.

Fullerene ligands, **L1** and **L2**, were synthesized as described in the experimental section. Although **L1** was reported in our previous work [23], the growth of single-component crystals is first reported here (Figure 1a). **L1** crystallizes in the *P*2/*c* space group with the asymmetric unit consisting of two halves of molecules. The crystal structure of **L1** confirmed that it is a hexa-adduct of C_60_ with two 4,5-diazafluorene groups (dinitrogen chelating ligands) located at the *trans*-1 position and four malonate groups located at the equatorial position of C_60_. Fullerene ligand **L2** (Figure 1b) was synthesized according to the procedure as described for **L1**. It is also a hexa-adduct of C_60_ with two *trans*-1 phenyl oxazole groups (N,O-chelating ligands) and four equatorial malonates groups. As is well known, transition metals have empty orbitals available for accepting electrons from O- and N-donors to form coordinate bonding, thus both of the fullerene ligands were used as building units to assemble with Zn(NO_3_)_2_·6H_2_O, Cd(NO_3_)_2_·4H_2_O, Cu(NO_3_)_2_·3H_2_O, Cu(OAc)_2_·H_2_O, FeCl_2_·4H_2_O and FeCl_3_·6H_2_O. Six one-dimensional (1D) metal–fullerene frameworks (MFFs) were successfully synthesized from **L1**. Unexpectedly, no crystalline polymeric structures or discrete complexes were obtained from **L2**, which can possibly be ascribed to the O-based groups with higher electronegativity and less donating property. **L1**, as a bidentate N-containing ligand, has been reported to assemble with AgSO_3_CF_3_ and AgBF_4_, forming a 1D coordination polymer with 4-coordinate silver centers, and a discrete fullerene complex with 6-coordinate silver centers, respectively [23]. The results indicate that metal salts play an important role in metal–fullerene polymeric structures. To further explore the influence of metal salt properties on MFFs, Zn(II)/Cd(II)/Cu(II) nitrates, Cu(II) acetate, as well as Fe(II)/Fe(III) chlorides, was selected to coordinate with **L1** under the same conditions. Single crystals of compounds **1**–**6** were grown from the same solvent combination, and their structures were studied in this work.

### 3.1. MFFs from Three Metal Nitrates: Crystal Structures of ZnL1(NO_3_)_2_(H_2_O)_2_ (***1***), CdL1(NO_3_)_2_ (***2***) and Cu(L1)(H_2_O)_2_(NO_3_)_2_ (***3***)

**Figure 2:**
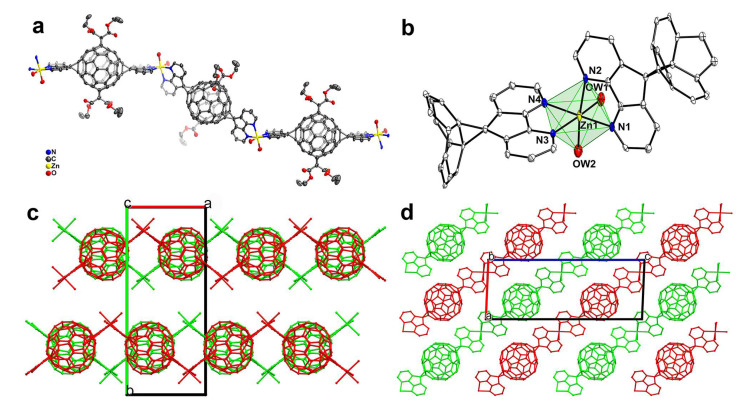
(**a**) A drawing of a portion of the 1D stair-like chain structure of compound **1** with thermal ellipsoids shown at 50%, front and back malonate addends and all hydrogen atoms have been omitted for clarity; (**b**) coordination environment of Zn(II) in **1** with 25% thermal contours; (**c**) packing mode of **1** along the *a*-axis; (**d**) ABAB-type alignment of individual layers. Hydrogen atoms and malonate addends have been omitted for clarity.

Three divalent transition metal nitrates, Zn(NO_3_)_2_·6H_2_O, Cd(NO_3_)_2_·4H_2_O and Cu(NO_3_)_2_·3H_2_O, were firstly assembled with **L1** to show the diverse supramolecular structures based on different metal centers. Crystals of **1**–**3** are described and compared together, since they are formed from nitrate salts with different divalent metal ions. Notably, the differences in the crystal structures should be caused by the unique aspects of the metal ions. Light yellow crystals of Zn**L1**(NO_3_)_2_(H_2_O)_2_ (**1**) were obtained by slow diffusion of Zn(NO_3_)_2_·6H_2_O in methanol into a solution of **L1** in dichloromethane. Compound **1** crystallized in the *P*2_1_/*c* space group and the asymmetric unit was composed of one molecule of **L1**, one Zn ion, and two water molecules. The assembly of **L1** with zinc ions formed an unexpected 1D stair-like chain structure (Figure 2a,b) instead of the expected linear chain as in the case of [Zn(dafo)_2_(H_2_O)_2_](NO_3_)_2_ (dafo = 4,5-diazafluoren-9-one) [29], which could be ascribed to the attachment of the fullerene cage on the diazafluorene in **L1**. The counter anions NO_3_^−^ are not connected with the Zn ions and are not observed in the structure, since they are in the disordered volume. This phenomenon has been previously reported [8]. Each fullerene unit coordinates with two zinc ions through two diazafluorene appendages. The local coordination environment of the zinc center, as shown in Figure 2b, is hexa-coordinated with four nitrogen atoms from two diazafluorene units and two oxygen atoms from two water (H_2_O) molecules. The bond distances between the zinc ion and the nitrogen/oxygen atoms are 2.131(6) Å for Zn1-N1, 2.245(7) Å for Zn1-N2, 2.280(8) Å for Zn1-N3, 2.134(6) Å for Zn1-N4, 2.115(9) Å for Zn1-O_w_1, and 2.089(9) Å for Zn1-O_w_2, respectively. The angles centered on the zinc ion are 93.1(3)° for O_W_1-Zn1-N1, 92.7(3)° for N1-Zn1-N3, 81.5(3)° for N3-Zn1-N4, and 93.2(3)° for N4-Zn1-O_W_1, respectively. The sum of these four angles is 360.5°, indicating that Zn1, N1, N2, N3 and O_W_1 are almost on the same plane. The angle of the N2-Zn1-O_W_2 axis is 174.0(3)°, which is very close to being linear. Therefore, the coordination geometry of the zinc center can be considered as a distorted octahedron (shown in green in Figure 2b). The stair-like chains are arranged in a staggered manner and repeated in every two adjacent chains, resulting in an ABAB-type stacking (Figure 2c,d).

**Figure 3:**
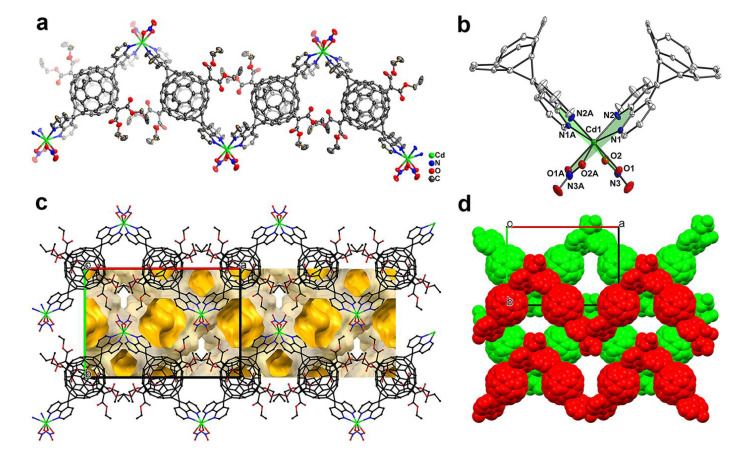
(**a**) The 1D infinite zigzag chain viewed down the *c*-axis in **2** with thermal ellipsoids shown at 50%, front and back malonate addends and all hydrogen atoms have been omitted for clarity; (**b**) the local environment of Cd1 in **2**; (**c**) voids shown in bright yellow occupying approximately 43 % of **2**; (**d**) interlayer relationship within the crystal lattice as seen down the *c*-axis. Hydrogen atoms and malonate addends have been omitted for clarity.

To investigate the effect of metal ions on the structure of MFFs, Cd(NO_3_)_2_·4H_2_O and Cu(NO_3_)_2_·3H_2_O were adopted to assemble with the **L1** ligand as well. Accordingly, the synthesis of compounds **2** and **3** are useful in comparing the possible structural changes that may occur due to the alternation of the metal ions. Yellow crystal of polymeric Cd(**L1**)(NO_3_)_2_ (**2**) was obtained by slow diffusion of Cd(NO_3_)_2_·4H_2_O in methanol into a solution of **L1** in dichloromethane. Compound **2** crystallized in the *I*2/*a* space group with the asymmetric unit consisting of half of molecule **L1**, half of Cd ion and one NO^3−^ counter anion. The structural examinations reveal that the connection of the Cd(II) and diazafluorene of **L1** forms a 1D zigzag chain, propagating with an approximately perpendicular folding angle in the crystallographic *a* direction (Figure 3a). Each Cd ion is octa-coordinated by four nitrogen atoms (Cd1–N1/N1A, 2.525(4) Å, and Cd1-N2/N2A, 2.369(4) Å) from two diazafluorenes of different fullerene linkers and four oxygen atoms (Cd1–O1/O1A, 2.317(4) Å, and Cd1-O2/O2A, 2.611(5) Å) from two nitrate anions. The octahedrally coordinated Cd ion exhibit *η*^2^ coordination to both nitrate anions. The sum of the bond angles of N1-Cd1-N2, N2-Cd1-O1A, O1A-Cd1-O2A and O2A-Cd1-N1 is 359.8°, suggesting that Cd1, N1, N2, O1A and O2A atoms are almost coplanar. Similarly, Cd1, N1A, N2A, O1 and O2 atoms are located on the same plane. The two planes crossed each other at the point of the Cd ion, presenting a butterfly-shaped structure (Figure 3b). Interestingly, large voids of ~5066 Å^3^, shown in bright yellow and occupying approximately 43% of the unit cell, are regularly distributed within and between the one-dimensional polymer chains (Figure 3c), suggesting the potential applications for solvent or gas absorption, isolation and storage. Each 1D zigzag chain runs one over the next in an ABAB sequence along the *c*-axis (Figure 3d).

**Figure 4:**
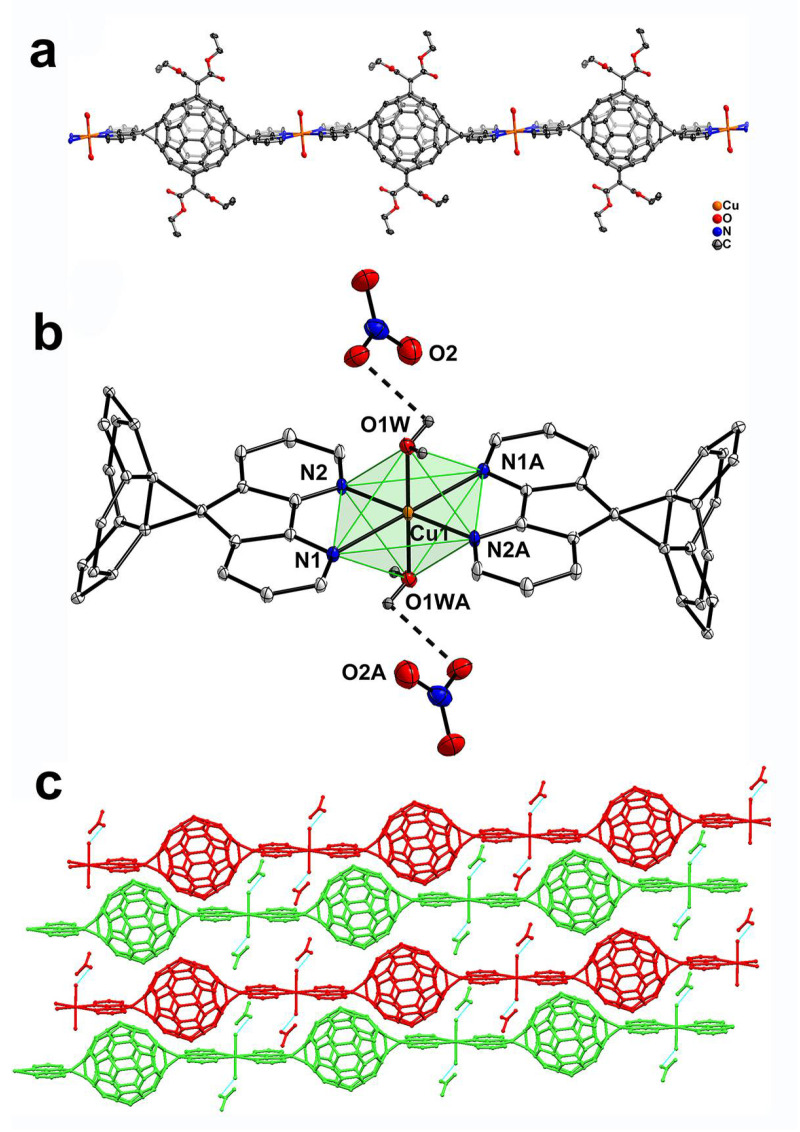
(**a**) 1D linear chain structure of Cu(**L1**)(H_2_O)_2_(NO_3_)_2_ (**3**), front and back malonate addends and all hydrogen atoms have been omitted for clarity; (**b**) the local environment of Cu center; (**c**) packing structure of **3**, hydrogen atoms and malonate addends have been omitted for clarity.

Interestingly, the reaction of **L1** with Cu(NO_3_)_2_ yielded the expected 1D straight line (Figure 4a) instead of the stair-like (Zn(II)-based MFF, **1**) or the zigzag (Cd(II)-based MFF, **2**) structure. Green crystals of Cu(**L1**)(H_2_O)_2_(NO_3_)_2_ (**3**) crystallized in the *P*-1 space group with the asymmetric unit making up of half **L1** molecule, half of Cu ion, one coordinated water, and a free NO^3−^ counter anion. The Cu ion is located at the crystallographic inversion center and is hexa-coordinated with four nitrogen atoms occupying the equatorial plane and two terminal H_2_O molecules on the axial position (Figure 4b). It is worth noting that the diazafluorene shows asymmetric chelation, with one Cu-N bond (2.557(2) Å) being much longer than the other one (1.998(2) Å). The sum of four equatorial angles around copper is exactly 360.0°, indicating a square-planar arrangement of the Cu-N_4_ unit. The O1-Cu1-O1A (180.0°, Cu-O distance of 1.981(2) Å) coordination perpendicular to the equatorial plane fills the vacant axial position and results in an octahedral geometry of the Cu center. Additionally, the free nitrate anion (NO_3_^−^) is linked to the coordinated H_2_O by a hydrogen bond (O_w_1-H1···O2) with a distance of 2.65(4) Å and a corresponding angle of 177.1(3)°. The linear chains run in parallel, and no intermolecular hydrogen bonding interactions were observed due to the large fullerene units (Figure 4c).

The coordination between H_2_O and Cu ion is not very strong, and could be substituted by a second building unit (SBU), such as di- or tetrapyridine-based pillars [30], which suggests that compound **3** stands out as an attractive building block for 2D and 3D metal–fullerene frameworks. Based on the above results, it can be concluded that the introduction of different metal ion centers accounts for the diversities of the structures. As a result, the polymeric structure has evolved from the 1D stair-like chain [Zn**L1**(NO_3_)_2_(H_2_O)_2_] to the 1D zigzag chain [Cd**L1**(NO_3_)_2_] and the 1D linear chain [Cu**L1**(H_2_O)_2_(NO_3_)_2_] due to the distinctive coordination mode of each metal ion.

### 3.2. MFFs from Copper Salts with Different Counter Anions: Crystal Structure of CuL1(OAc)(CH_3_O) (***4***)

Our previous studies on the reactions of **L1** with AgSO_3_CF_3_ and AgBF_4_ showed the dependence of coordination modes and structures of MFFs on the counter anions [27]. Therefore, we take Cu(II) acetate [Cu(OAc)_2_·H_2_O] as a contrast to Cu(NO_3_)_2_ to further survey the structure regulation by counter anions.

**Figure 5:**
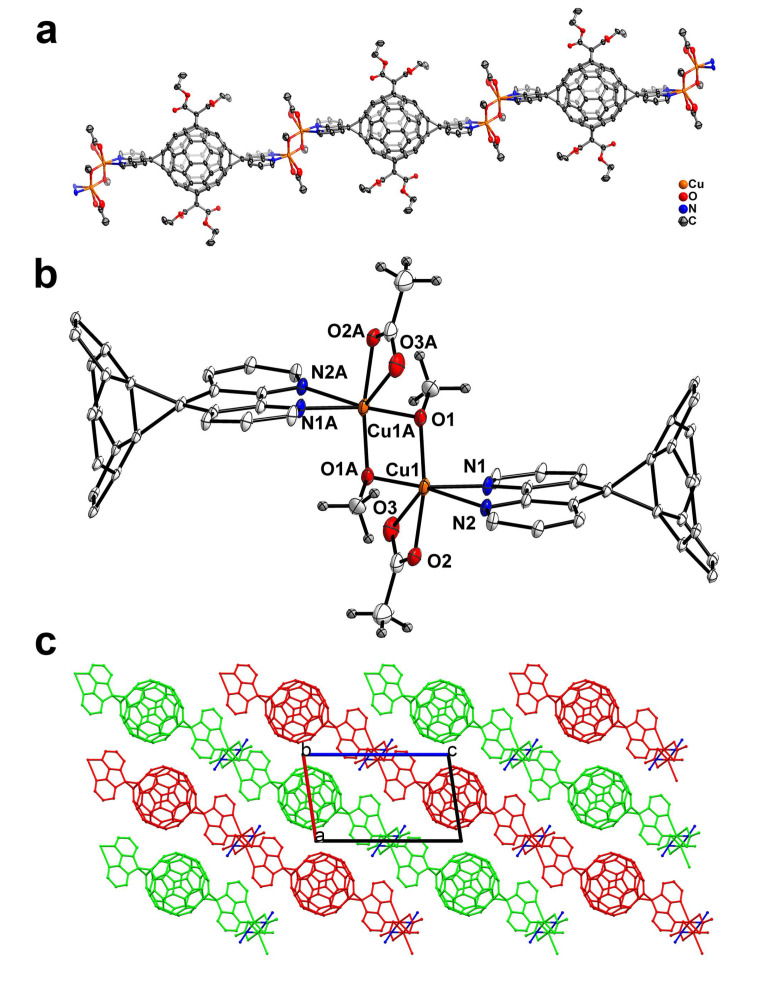
Perspective view of (**a**) 1D dinuclear Cu(II)-linked stair-like chain structure of **4**, front and back malonate addends and all hydrogen atoms have been omitted for clarity; (**b**) the coordination environment of Cu ions in **4**; (**c**) the parallel packing motif of **4** viewed along the *b-*axis; hydrogen atoms and malonate addends have been omitted for clarity.

The reaction of **L1** and Cu(OAc)_2_·H_2_O produced green crystals of compound **4** with the composition of Cu**L1**(OAc)(CH_3_O). The space group of **4** was determined to be *P*-1, which is the same as compound **3**. Its asymmetric unit contains half of molecule **L1**, one Cu ion, one CH_3_COO^−^ anion and one CH_3_O^−^ anion. Unexpectedly, the polymeric structure of compound **4** is a 1D stair-like chain (Figure 5a) instead of a 1D straight line as shown in compound **3**. The fullerene units in **4** are unprecedentedly connected by a bimetallic Cu(II) center (Figure 5b) instead of a single Cu(II) as presented in crystal **3**. Further crystallographic analysis reveals that Cu1 and Cu1A possess the identical coordination environments of two nitrogen atoms from one diazafluorene unit (Cu–N distances of 2.013(3) Å and 2.630(2) Å), two oxygen atoms from one acetate anion, and two bridging oxygen from CH_3_O^−^ anions generated from methanol (a solvent for crystallization). The coordinated Cu ions exhibit an *η*^2^ coordination mode to the acetate anions. The distance of Cu1-Cu1A is 2.992(9) Å, longer than the van der Waals contact (2.8 Å) for Cu-Cu [31,32], suggesting that no metal-metal bond is formed between the two copper ions. To the best of our knowledge, compound **4** represents the first example of a dinuclear Cu(II)-linked fullerene coordination polymer. The packing mode of **4** is shown in Figure 5c.

The use of Cu(NO_3_)_2_ and Cu(OAc)_2_ as the building blocks led to different coordination configurations of the Cu centers and accordingly the different polymeric structures. Specifically, the NO_3_^−^ anion was not coordinated with the Cu ion in compound **3**, thereby facilitating the addition of two fullerene linkers on one Cu ion. Meanwhile, the CH_3_COO^−^ anion, as a bidentate chelating ligand, binds to the Cu ion in **4**, preventing the coordination of another diazafluorene unit with the same Cu ion. As a result, the structure was adjusted from a monometallic 1D linear chain to a bimetallic 1D stair-like chain upon changing the counter anions. In addition, it is worth noting that compound **4** is the only metal–fullerene framework in which the metal center is coordinated with only one fullerene ligand.

### 3.3. MFFs from Iron Chloride with Different Oxidation States: Crystal Structures of FeL1Cl_2_ (***5***) and FeL1Cl_2_(FeCl_4_) (***6***)

Heretofore, metal atoms from Group IB (Cu and Ag) [23] and Group IIB (Zn and Cd) have been studied for supramolecular assembly with fullerene ligand **L1**. Each metal ion exhibits distinct coordination configurations. Notably, in addition to Group IB and IIB metal elements, Group VIII elements, such as iron, are excellent candidates to coordinate with **L1**. A more important reason for picking iron salts is that they have two oxidation states, making it possible for us to seek the effect of the oxidation states in preparing MFF structures.

**Figure 6:**
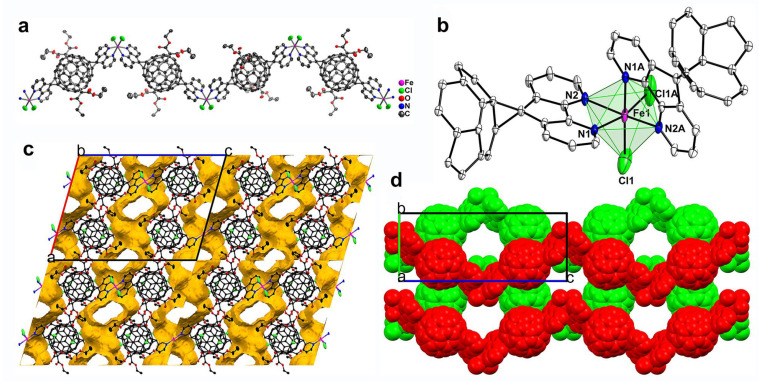
(**a**) 1D zigzag chain structure viewed down the *a*-axis in **5**. Front and back malonate addends and all hydrogen atoms were omitted for clarity; (**b**) coordination environment of Fe(II) cation; (**c**) voids between chains shown along the *b-*axis; (**d**) interlayer relationship within the crystal lattice as seen from the *a*-axis; hydrogen atoms and malonate addends have been omitted for clarity.

Bulk green crystals of Fe**L1**Cl_2_ (**5**) were grown by slow diffusion of methanol solution of FeCl_2_·4H_2_O into a dichloromethane solution of **L1**. Compound **5** crystallized in the *I*2/*a* space group with the asymmetric unit containing half of molecule **L1**, half of Fe(II) ion, one Cl^−^ anion, and one CH_2_Cl_2_ molecule. The structural analysis shows that the packing mode of compound **5** is analogous to that of compound **2**, forming a 1D zigzag chain along the *c-*axis, which runs one over the next in parallel and stacks in an ABAB sequence along the *b*-axis (Figure 6a,d). The central Fe(II) is hexa-coordinated by two chlorine atoms with a Fe-Cl distance of 2.356(3) Å, and four nitrogen atoms from two diazafluorene units of **L1** with the Fe-N distances of 2.214(3) Å and 2.269(2) Å (Figure 6b). The four angles around the central Fe(II) are 87.98(9)° for N1-Fe1-N2A, 100.54(1)° for N2A-Fe1-Cl1A, 90.69(1)° for N2-Fe1-Cl1A, and 79.62(9)° for N1-Fe1-N2, forming a slightly deviated plane. Additionally, the angle of Cl1-Fe-N1A across the plane is 166.79(11)°, resulting in a severely deformed octahedron coordination geometry for the central Fe(II) ion. Notably, as shown in Figure 6c, the voids of ~2037 Å^3^, occupying approximately 18% of the unit cell, stagger front and rear with the one-dimensional chains.

**Figure 7:**
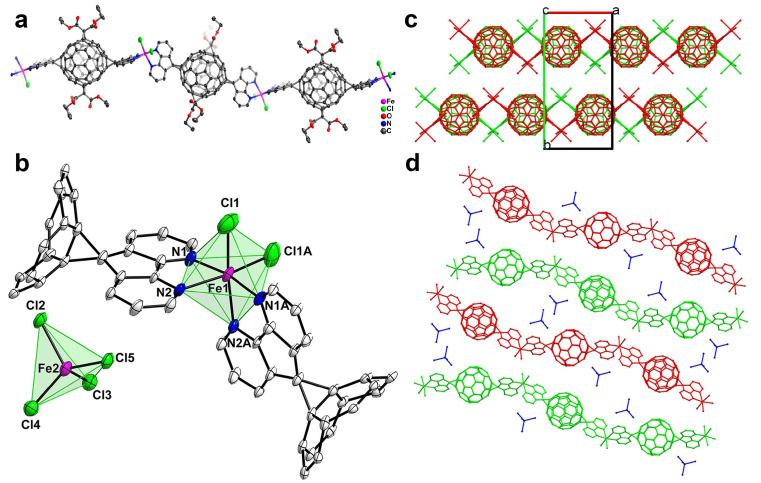
(**a**) 1D polymer chain structure in **6**. Front and back malonate addends and all hydrogen atoms were omitted for clarity; (**b**) coordination environments of Fe(III) cations; (**c**) the details of packing structure viewed down the *c*-axis; (**d**) ABAB-type alignment of individual chains; all the hydrogen atoms and malonate addends have been omitted for clarity.

As a comparison, trivalent FeCl_3_·6H_2_O was adopted to assemble with **L1** as well. The reaction of FeCl_3_·6H_2_O and **L1** generated red crystals of Fe(**L1**)Cl_2_(FeCl_4_) (**6**). As evidenced by the single-crystal structural analysis, compound **6** has the *I*2/*a* space group, the same as that of compound **5**. The asymmetric unit consists of half of the **L1** molecule, half of Fe(III) ion, one Cl^−^ anion and half of [FeCl_4_]^−^ counter anion. Figure 7a shows that a 1D chain structure is formed, which is different from the zigzag shape in compound **5**. The packing mode as displayed in Figure 7c,d is also different from that of compound **5**. The central Fe(III) ion is hexa-coordinated by two chlorine atoms with a Fe-Cl distance of 2.333(5) Å, and four nitrogen atoms from two diazafluorene units with the Fe-N distances of 2.238(6) Å and 2.242(7) Å, which is similar to the coordination of the central Fe1(II) ion in compound **5**. The sum of the four angles around the central Fe(III) ion is 359.97° (N1A-Fe1-Cl1A, 96.93(2)°; N1A-Fe1-N2, 93.07(2)°; N2-Fe1-N1, 80.03(2)°; N1-Fe1-Cl1A, 89.94(2)°), and the angle of Cl1-Fe1-N2A is 169.97(2)°, forming a deformed octahedron coordination geometry as observed in **5**. However, the presence of Fe^3+^ makes a unit of [FeCl_4_]^−^ appear as a counter anion in the crystal to stabilize compound **6** (Figure 7b). All the [FeCl_4_]^−^ units occupy the spaces between the 1D chains (Figure 7d), and the central Fe2(III) ion is bound by four chlorine atoms with Fe-Cl distances in the range of 2.114(4)-2.189(4) Å, in agreement with the reported values for Fe-Cl bonds [33].

## 4. Discussion

Based on the above studies, metal salts exhibit remarkable capabilities to regulate the structures of metal–fullerene frameworks. For compounds **1**, **2** and **3**, prepared from different metal nitrates (Zn(NO_3_)_2_, Cd(NO_3_)_2_ and Cu(NO_3_)_2_), Zn and Cu ions exhibited a stronger affinity for H_2_O rather than the multidentate ligand NO_3_^−^, while Cd ions preferred to coordinate with NO_3_^−^ instead of H_2_O. Surprisingly, when Cu(NO_3_)_2_ was replaced by Cu(OAc)_2_, the ligands of Cu(II) were accordingly changed from H_2_O in **3** to CH_3_O^−^ and AcO^−^ anions in **4**. Compounds **5** and **6** were synthesized from iron chlorides with different valence states. Both Fe(II) and Fe(III) ions are inclined to bind monodentate Cl^−^ rather than H_2_O or CH_3_O^−^ anions. These results could be interpreted as the preferred coordination modes of different metal ions, including coordination ability toward the ligands, coordination number, and coordination geometry.

## 5. Conclusions

In summary, six new 1D metal–fullerene frameworks (**1**–**6**) based on **L1** and different metal salts were successfully synthesized, and their structures were unambiguously confirmed by single-crystal diffraction. The viability of **L1** in fabricating fullerene-based supramolecular architectures was established. The structural diversity of compounds **1** to **6** revealed the regulation of metal salts on the MFF structures. The methodical changes to the metallic building block delivered more knowledge of fullerene coordination chemistry, and more examples were added to the limited database available on crystalline metal–fullerene frameworks. Three factors in the coordination chemistry of MFFs were reviewed: the metal cation, the counter anion, and the oxidation state of the metal. The distinct coordination modes and coordination numbers of each metal ion (Zn^2+^, Cd^2+^, Cu^2+^), the different coordination abilities of counter anions (NO_3_^−^, AcO^−^) to metal centers and the presence of [FeCl_4_]^−^ units due to the higher oxidation state account for the great structural changes of MFFs. Interestingly, compound **3** may become a promising building unit for 2D and 3D MFFs if the coordinated waters are replaced by di- or tetrapyridine-based pillars. Compound **4** is the first example of a dinuclear metal-linked MFF. This study not only contributes to the basic science of MFFs, but is also valuable for the design and synthesis of next-generation multi-dimensional metal–fullerene frameworks.

## Data Availability

The data presented in this study are available on a reasonable request from the corresponding author.

## Supplementary Materials

The following supporting information can be downloaded at: https://www.mdpi.com/article/10.3390/nano12081314/s1, Figure S1: (a) ^1^H NMR spectrum of **L2** (600 MHz, CDCl_3_) and expanded parts: (b) 5.50–4.10 ppm, and (c) 1.30–1.20 ppm, R = [C-(OOC_2_H_5_)_2_]; Figure S2: ^13^C NMR spectrum of L2 (150 MHz, CDCl3). Figure S3. Photographs of the crystals 1–6. Table S1: Crystal Data and Structure Refinements for Compounds **L1** and **1**–**6**.
