# Supplementary material for: Diversity of Metal—Fullerene Framework Structures Regulated by Metal Salts
Source: Nanomaterials (Basel). 2022 Apr 12;12(8):1314. doi: 10.3390/nano12081314 (PMC9025373; doi:10.3390/nano12081314)
Supplement: Supplementary file 1 [file nanomaterials-12-01314-s001.zip › nanomaterials-1557317-supplementary.pdf]

Supporting Information

Diversity of Metal-Fullerene Framework Structures Regulated by Metal Salts

Jingjing Wang [1,†], Yang-Rong Yao [2,†], Shaoting Yang [1], Xinyi Zhou [1], Ao Yu [1], Ping Peng [1,*] and Fang-Fang Li [1,*]

[1] State Key Laboratory of Materials Processing and Die & Mould Technology, School of Materials Science and Engineering, Huazhong University of Science and Technology, Wuhan 430074, China; Jjwangup@163.com (J.W.); hust_yst@163.com (S.Y.); zhou99xy@163.com (X.Z.); aoyuhust@hust.edu.cn (A.Y.)

[2] Department of Chemistry, University of Texas at El Paso, El Paso, TX 79968, USA; yyr880821@163.com

* Correspondence: ppeng@hust.edu.cn (P.P.); ffli@hust.edu.cn (F.-F.L.)

† These authors contributed equally to this work.

Citation: Wang, J.; Yao, Y.-R.; Yang, S.; Zhou, X.; Yu, A.; Peng, P.; Li, F.-F. Diversity of Metal–Fullerene Framework Structures Regulated by Metal Salts. *Nanomaterials* **2022**, *12*, 1314. http://doi.org/10.3390/nano12081314

Academic Editor(s): Placido Mineo

Received: 30 December 2021
Accepted: 20 January 2022
Published: 12 April 2022

Publisher's Note: MDPI stays neutral with regard to jurisdictional claims in published maps and institutional affiliations.

Copyright: © 2022 by the authors. Submitted for possible open access publication under the terms and conditions of the Creative Commons Attribution (CC BY) license (https://creativecommons.org/licenses/by/4.0/).

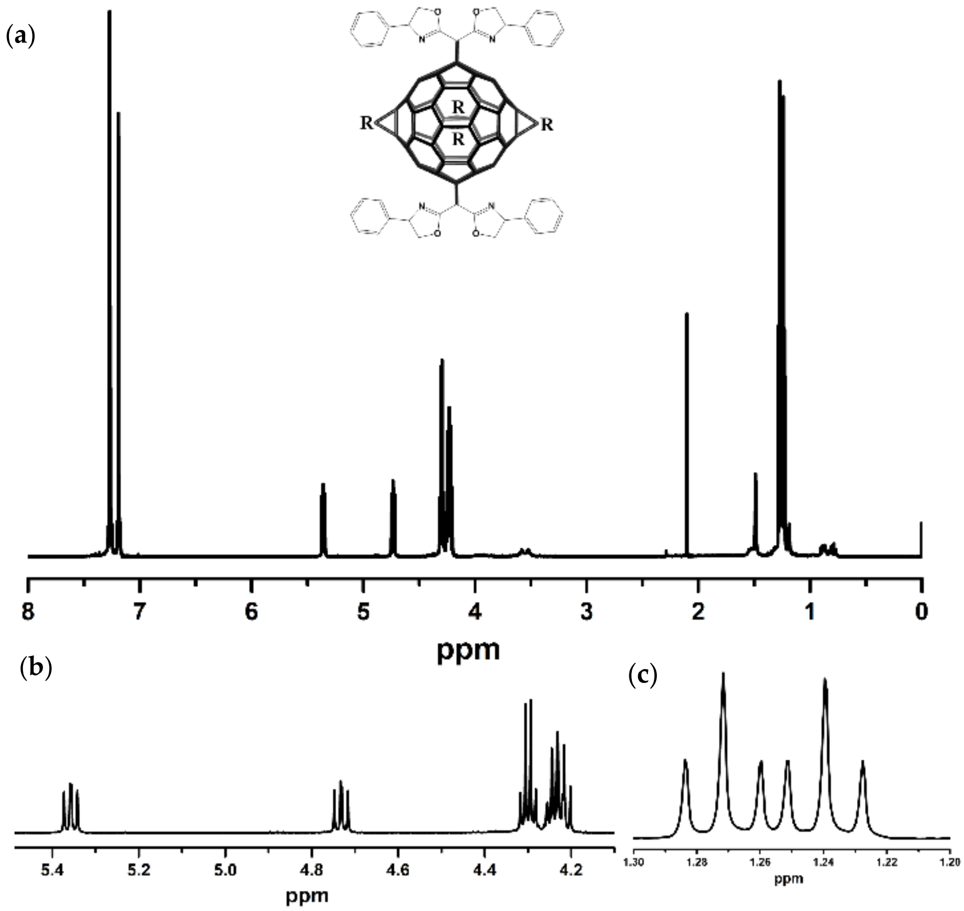

Figure S1. (**a**) ^{1}H NMR spectrum of **L2** (600 MHz, $CDCl_3$) and expanded parts: (**b**) 5.50–4.10 ppm, and (**c**) 1.30–1.20 ppm, R = [C-$(OOC_2H_5)_2$]

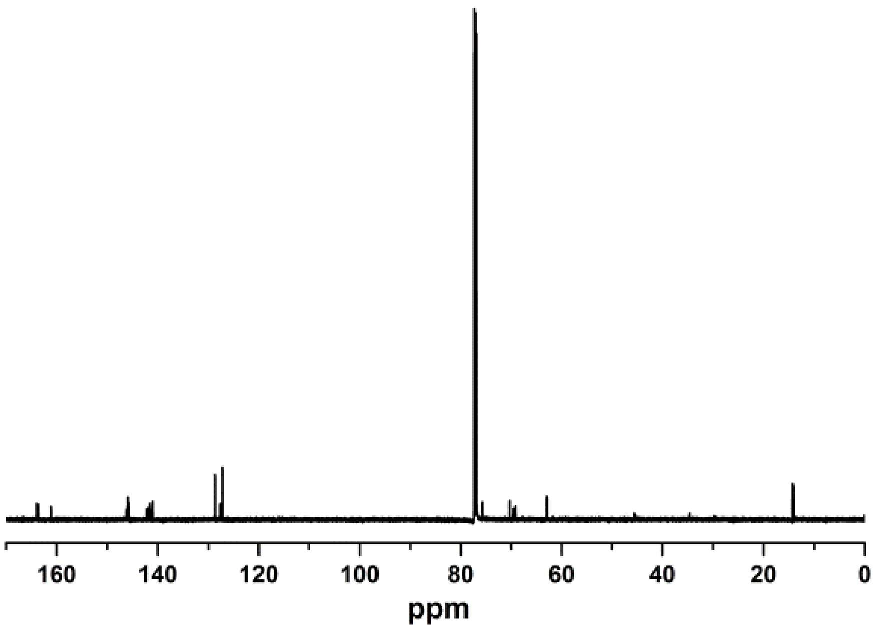

Figure S2. ^{13}C NMR spectrum of **L2** (150 MHz, $CDCl_3$).

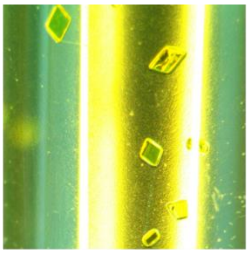

$ZnL1(NO_3)_2(H_2O)_2$ (1)

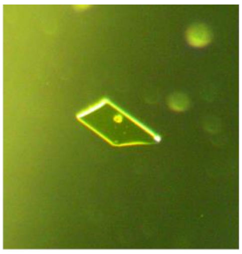

$CdL1(NO_3)_2$ (2)

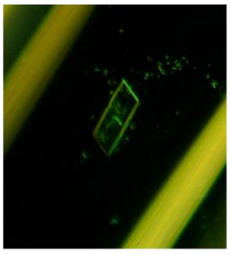

$CuL1(H_2O)_2(NO_3)_2$ (3)

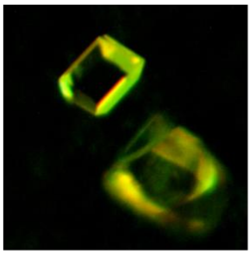

$CuL1(OAc)(CH_3O)$ (4)

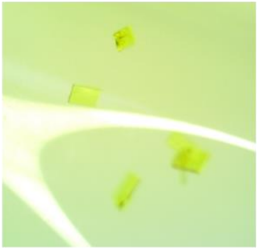

$FeL1Cl_2$ (5)

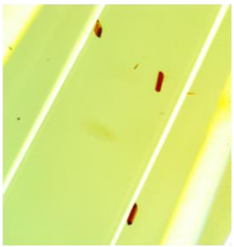

$FeL1Cl_2(FeCl_4)$ (6)

Figure S3. Photographs of the crystals **1**–**6**.

Table S1. Crystal Data and Structure Refinements for Compounds **L1** and **1–6**.

compound	**L1**	**1**	**2**	**3**	**4**	**5**	**6**
empirical formula	$C_{110}H_{12}N_4O_{16}$	$C_{110}H_{56}N_4O_{18}Zn$	$C_{110}H_{52}CdN_6O_{22}$	$C_{110}H_{56}CuN_6O_{24}$	$C_{116}H_{64}Cu_2N_4O_{22}$	$C_{110}H_{56}Cl_6FeN_4O_{16}$	$C_{110}H_{52}Cl_6Fe_2N_4O_{16}$
formula weight	1685.55	1786.95	1921.97	1909.14	1992.79	1982.15	2009.95
color	yellow	yellow	yellow	green	green	green	red
crystal size (mm^3)	0.2×0.15×0.05	0.08×0.08×0.02	0.3×0.15×0.05	0.2×0.08×0.03	0.15×0.1×0.05	0.1×0.08×0.06	0.1×0.08×0.05
crystal system	monoclinic	monoclinic	monoclinic	triclinic	triclinic	monoclinic	monoclinic
space group	$P2/c$	$P2_1/c$	$I2/a$	P-1	P-1	$I2/a$	$I2/a$
a (Å)	27.0660(6)	11.0789(9)	27.0491(12)	10.6736(2)	10.7271(2)	25.4056(4)	11.8240(11)
b (Å)	10.8766(2)	26.346(3)	18.3914(9)	13.6605(2)	14.0625(3)	13.3162(1)	23.642(2)
c (Å)	27.4896(7)	29.197(3)	24.6629(13)	16.6244(3)	16.9325(3)	34.1197(5)	30.612(2)
α (deg)	90.0	90.0	90.0	92.400(1)	78.875(2)	90.0	90.0
β (deg)	111.788(3)	92.426(2)	103.757(5)	91.712(1)	78.223(2)	105.807(2)	92.591(8)
γ (deg)	90.0	90.0	90.0	109.767(1)	68.646(2)	90.0	90.0
V (Å^3)	7514.4(3)	8514.7(14)	11917.1(10)	2276.62(7)	2309.04(9)	11106.4(3)	8548.6(13)
Z	4	4	4	1	1	4	4
Dx (g/cm^3)	1.490	1.394	1.071	1.393	1.433	1.185	1.562
R_{int}	0.044	0.1338	0.0984	0.0348	0.0492	0.0337	0.0547
R_1 [$I \geq 2\sigma(I)$]	0.0676	0.1284	0.0835	0.0556	0.0705	0.0662	0.0898
wR_2 (all data)	0.1968	0.3034	0.2307	0.1685	0.2053	0.2014	0.2616
completeness	0.972	0.946	1.000	0.997	0.996	0.981	0.984
GOF	1.036	1.041	1.110	1.064	1.075	1.073	1.063
CCDC	1878371	1878374	1878376	1878377	1878378	1878370	1878379

$R_1=\sum||F_o|-|F_c||/\sum|F_o|$, $wR_2=[\sum w(F_o^2-F_c^2)^2]/\sum w(F_o^2)^2]^{1/2}$
